# Giant cell arteritis: incidence and phenotypic distribution in Western Norway 2013–2020

**DOI:** 10.3389/fmed.2023.1296393

**Published:** 2023-12-12

**Authors:** H. K. Skaug, B. T. Fevang, J. Assmus, A. P. Diamantopoulos, G. Myklebust, L. K. Brekke

**Affiliations:** ^1^Haugesund Hospital for Rheumatic Diseases, Haugesund, Norway; ^2^Department of Clinical Science (K2), Faculty of Medicine, University of Bergen, Bergen, Norway; ^3^Department of Rheumatology, Bergen Group of Epidemiology and Biomarkers in Rheumatic Disease (BEaBIRD), Haukeland University Hospital, Bergen, Norway; ^4^Center for Clinical Research, Haukeland University Hospital, Bergen, Norway; ^5^Division of Internal Medicine, Department of Infectious Diseases, Akershus University Hospital, Lørenskog, Norway; ^6^Research Department, Hospital of Southern Norway, Kristiansand, Norway

**Keywords:** vasculitis, large vessel, giant cell arteritis, temporal arteritis, phenotypes, epidemiology, incidence, clinical features

## Abstract

**Objectives:**

There is an increasing awareness of the spectrum of phenotypes in giant cell arteritis (GCA). However, there is sparse evidence concerning the phenotypic distribution which may be influenced by both genetic background and the environment. We established a cohort of all GCA-patients in the Bergen Health Area (Western Norway), to describe the phenotypic distribution and whether phenotypes differ with regards to incidence and clinical features.

**Methods:**

This is a retrospective cohort study including all GCA-patients in the Bergen Health Area from 2013–2020. Data were collected by reviewing patient records, and patients considered clinically likely GCA were included if they fulfilled at least one set of classification criteria. Temporal artery biopsy (TAB) and imaging results were used to classify the patients according to phenotype. The phenotype “cranial GCA” was used for patients with a positive TAB or halo sign on temporal artery ultrasound. “Non-cranial GCA” was used for patients with positive findings on FDG-PET/CT, MRI-, or CT angiography, or wall thickening indicative of vasculitis on ultrasound of axillary arteries. Patients with features of both these phenotypes were labeled “mixed.” Patients that could not be classified due to negative or absent examination results were labeled “unclassifiable”.

**Results:**

257 patients were included. The overall incidence of GCA was 20.7 per 100,000 persons aged 50 years or older. Overall, the cranial phenotype was dominant, although more than half of the patients under 60 years of age had the non-cranial phenotype. The diagnostic delay was twice as long for patients of non-cranial and mixed phenotype compared to those of cranial phenotype. Headache was the most common clinical feature (78% of patients). Characteristic clinic features occurred less frequently in patients of non-cranial phenotype compared to cranial phenotype.

**Conclusion:**

The overall incidence for GCA was comparable to earlier reports from this region. The cranial phenotype dominated although the non-cranial phenotype was more common in patients under 60 years of age. The diagnostic delay was longer in patients with the non-cranial versus cranial phenotype, indicating a need for examination of non-cranial arteries when suspecting GCA.

## Introduction

Giant cell arteritis (GCA) is a heterogeneous disease predominantly affecting women and almost exclusively persons over the age of 50 years (1). A pathogenic hallmark of GCA is wall inflammation of large and medium-sized arteries, but the underlying etiology is unclear (2). The triad of headache, jaw claudication, and visual disturbances has historically been viewed as a clinical hallmark (3). However, already in 1932 Horton et al. described an atypical variant with absence of peripheral pulses (4). Temporal artery biopsy (TAB) has long been considered as the gold standard in diagnosing GCA. Still, current recommendations suggest that a diagnosis also can be established based on strong clinical suspicion with positive imaging results (5). New imaging techniques have been developed and shown useful in the diagnostic process of GCA (6–9), and vascular imaging has been widely adopted in GCA (10, 11).

In recent decades there has been a growing interest in non-cranial GCA, also termed extra-cranial or large vessel GCA (LV-GCA). Still, no standardized classification of disease phenotypes exists. Studies on GCA phenotypes have mainly applied a binary division between cranial and non-cranial phenotype though acknowledging that some patients have a combination of the two (12–15), while a few recent studies have incorporated overlapping phenotypes (10, 16). Some authors have proposed that the disease spectrum also encompasses polymyalgia rheumatica (PMR) (1, 17).

This study included all GCA-patients diagnosed in the Bergen Health Area (Western Norway) from 2013 to 2020. Brekke et al. found that the incidence of GCA in the same area increased from 1972–1992 but remained stable from 1993–2012 (18, 19). However, imaging data were unavailable for the vast majority of patients diagnosed from 1972–2012, and < 1% of the GCA-patients had documented involvement of large arteries. The aim of the current study is to describe the phenotypic distribution in GCA and whether phenotypes differ with regards to incidence and clinical features.

## Materials and methods

### Study design and geographic setting

This is a retrospective cohort study including all GCA-patients diagnosed from 1 January 2013 to 31 December 2020 in the Bergen Health Area (BHA) in Western Norway. BHA serves a population of around 465.000. An overwhelming proportion of the population are Caucasian, and all other ethnicities represent minorities in this region. The only rheumatological department is located at Haukeland University Hospital in Bergen, and patients with suspected vasculitis are referred there, although sometimes via other departments at the hospital, such as the department of ophthalmology.

### Patient selection

Patients were identified by the diagnostic coding in the hospital register. All patients receiving in- or out-patient health care in a Norwegian hospital are assigned at least one diagnostic code from the International Classification of Diseases (ICD) on discharge. The ICD version 10 (ICD-10) was used for the entire study period. For the initial patient selection, we used the ICD-10-codes M31.5 “Giant cell arteritis with polymyalgia rheumatica,” M31.6 “Other giant cell arteritis,” and I77.6 “Arteritis, unspecified.” Patient records were reviewed, and data were recorded electronically. Cases were registered as clinically likely GCA if the following criteria were met: (1) the treating physician(s), according to patient records, considered GCA as the most likely diagnosis and chose to treat thereafter, and (2) the reviewing physician agreed that GCA was the most likely diagnosis. Among patients with clinically likely GCA, only those fulfilling at least one of the following sets of classification criteria were included: the American College of Rheumatology 1990 (ACR 1990) (20), the modified ACR 1990 proposed by Dejaco et al. (1), or the 2022 classification criteria from the American College of Rheumatology and the European Alliance of Associations for Rheumatology (ACR/EULAR 2022) (21).

### Collected variables and phenotype definitions

Data were collected according to a custom-made Excel template (Supplementary material). Date of symptom onset was registered when the uncertainty was maximum one month, otherwise it was registered as missing. Symptoms and clinical findings at the time of diagnosis were registered as present if they were noted to be present in the patient records, otherwise they were assumed to be absent. Laboratory values were registered if analyses were performed before treatment initiation. Missing laboratory results were registered as missing data. The variables regarding the results of TAB and imaging examinations [vascular ultrasound, computed tomography (CT), magnetic resonance imaging (MRI), and fluorodeoxyglucose positron emission tomography (FDG-PET)] were registered as missing if the examinations were not performed or the results were unavailable.

Imaging findings were regarded as positive if the radiologist described a thickening of the arterial wall compatible with vasculitis or, in the case of FDG-PET/CT, if the nuclear radiologist described FDG-uptake in the arterial wall compatible with vasculitis. Evaluated arteries included the thoracic and abdominal aorta, subclavian arteries, brachiocephalic trunk, axillary arteries, carotid arteries, and vertebral arteries, and in some cases common iliac arteries and proximal parts of the internal and external iliac arteries.

We defined three phenotypes of GCA according to the results of TAB and imaging diagnostics. The phenotype “cranial GCA” was used for patients with a positive TAB or halo sign on temporal artery ultrasound. “Non-cranial GCA” was used for patients with positive findings on FDG-PET/CT, MRI-, or CT angiography, or wall thickening indicative of vasculitis on ultrasound of axillary arteries. Patients with features of both these phenotypes were labeled “mixed.” Patients that could not be classified due to negative or absent examination results were labeled “unclassifiable”.

### Statistical analysis

Data registration was performed in Microsoft Excel and all data preparation, analysis, and visualization was done in R (22, 23). Descriptive statistics are presented as counts and proportions for discrete variables, whereas continuous variables are presented as median with interquartile range (IQR).

Using GCA patients ≥50 years of age and the corresponding background population, we estimated annual cumulative incidence and corresponding 95% confidence intervals by an exact Poisson method. Population data were acquired from Statistics Norway.^1^ Cumulative incidence, i.e., number of cases divided by population at risk, was calculated for each year, both in total and stratified by age group (<60 years, 60–69 years, 70–79 years, and 80+ years), sex, and phenotype.

We tested for association between phenotype and the following variables: sex, age group, diagnostic delay, and levels of C-reactive protein (CRP) and erythrocyte sedimentation rate (ESR) before treatment. We also tested for association between phenotype and the presence of different clinical characteristics. Depending on sample size, Chi square test or Fisher’s exact test was applied for categorical variables, while Kruskal-Wallis rank sum test was applied for continuous variables. Significance level (α) was set to 0.05. Altogether 19 significance-tests were performed, thus requiring the calculation of an adjusted α, corrected for multiple testing. The Bonferroni method, i.e., dividing α by the number of tests, gave an adjusted α of 0.0026. As the Bonferroni method is known to be conservative we also calculated the adjusted α by the less conservative Benjamini-Hochberg procedure (24), which gave an adjusted α of 0.018.

### Ethical considerations

The study was approved by the Regional Committee for Medical and Health Research Ethics (REC) (reference number REK-Vest 264,780). REC permitted access to patient records without obtained consent as it was considered that the participants’ integrity was sufficiently protected to grant this exemption in accordance with Norwegian law. We evaluated possible impacts of the data handling for the included patients through the preparation of a data protection impact assessment (DPIA).

## Results

### Patient characteristics and classification

**Figure 1:**
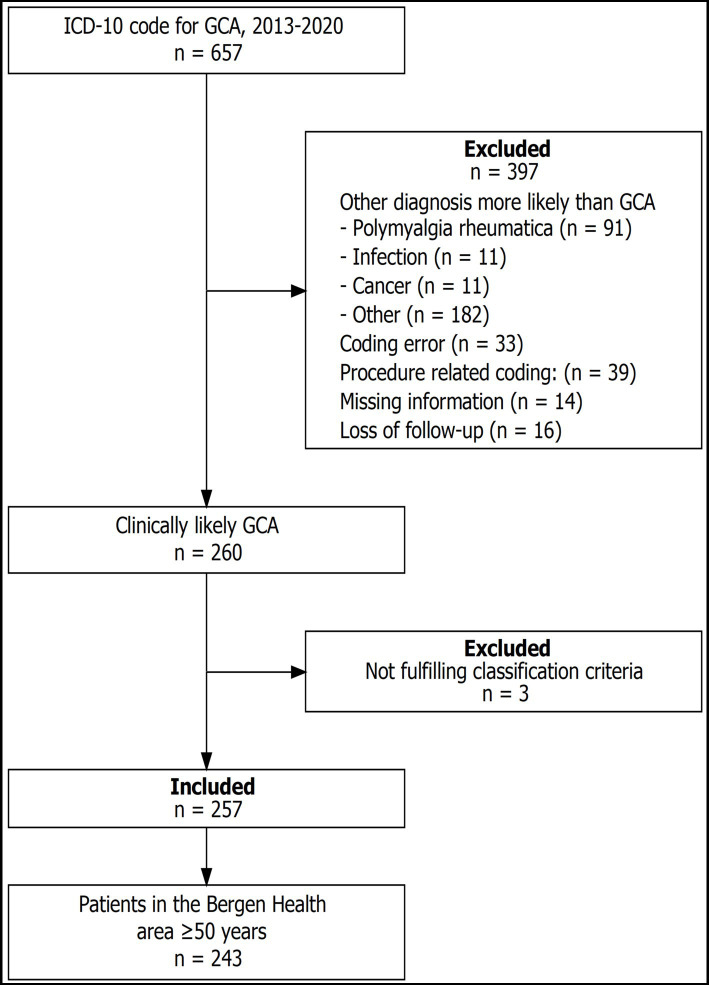
Flowchart showing the inclusion and exclusion of patients.

The cohort comprises 257 patients (Figure 1). Table 1 shows characteristics according to GCA phenotype, and the cranial phenotype was dominant. Time from symptom onset to diagnosis was longer for patients with non-cranial and mixed phenotype (*p* < 0.001) and the ESR (*p* < 0.001) was highest for patients with non-cranial phenotype (Table 1). Overall, more than 90% of the patients had undergone TAB. However, the proportion decreased in the last years of the study period. In 2018, 88% of the patients had undergone TAB, compared to 85% in 2019, and 76% in 2020. Similarly we observed an increasing proportion of patients who were examined by temporal artery ultrasound without having performed a TAB. For 2018 this constituted 5% of the patients, in 2019 12%, and in 2020 15%. The proportion of patients having undergone diagnostic imaging of non-cranial vessels increased throughout the study period from 18% in 2013 to 76% in 2020. Among all the patients only three had neither undergone biopsy nor imaging diagnostics.

**Table 1:** Cohort characteristics and overview of diagnostic procedures.

	Phenotype				
	Cranial *N* = 159	Non-cranial *N* = 41	Mixed *N* = 19	Unclassifiable *N* = 38	Total *N* = 257
Female sex	108 (68%)	30 (73%)	12 (63%)	31 (82%)	181 (70%)
Age at diagnosis^1^	74 (69, 81)	66 (58, 73)	66 (65, 74)	70 (63, 79)	72 (66, 79)
CRP before treatment^1^	71 (40, 115)	86 (62, 117)	61 (42, 108)	54 (33, 84)	70 (41, 114)
ESR before treatment^1^	78 (57, 95)	105 (74, 110)	89 (73, 100)	66 (40, 98)	80 (56, 101)
Days from symptoms to diagnosis^1^	40 (20, 94)	82 (59, 170)	80 (60, 116)	34 (15, 64)	51 (22, 96)
Any imaging performed	66 (42%)	41 (100%)	19 (100%)	18 (47%)	144 (56%)
Temporal artery biopsy					
Performed	155 (97%)	29 (71%)	16 (84%)	35 (92%)	235 (91%)
Positive	152 (96%)	0	14 (74%)	0	166 (65%)
Giant cells in biopsy	118 (74%)	0	8 (42%)	0	126 (49%)
Vascular ultrasound					
Performed^2^	52 (33%)	16 (39%)	11 (58%)	13 (34%)	92 (36%)
Axillary arteries examined	13 (8.2%)	9 (22%)	8 (42%)	2 (5.3%)	32 (12%)
Positive ultrasound^2^	41 (26%)	2 (4.9%)	9 (47%)	1 (2.6%)	53 (21%)
Halo in temporal artery	37 (23%)	0	8 (42%)	0	45 (18%)
Bilateral axillary involvement	0	2 (4.9%)	5 (26%)	0	7 (2.7%)
CT					
Performed^3^	7 (4.4%)	20 (49%)	9 (47%)	1 (2.6%)	37 (14%)
CT angiography	6 (3.8%)	6 (15%)	7 (37%)	0	19 (7.4%)
CT positive	0	13 (32%)	6 (32%)	0	19 (7.4%)
MR angiography					
Performed	6 (3.8%)	13 (32%)	3 (16%)	2 (5.3%)	24 (9.3%)
Positive	0	7 (17%)	3 (16%)	0	10 (3.9%)
Bilateral axillary involvement	0	0	0	0	0
FDG-PET/CT					
Performed	9 (5.7%)	35 (85%)	13 (68%)	3 (7.9%)	60 (23%)
Positive	0	35 (85%)	13 (68%)	0	48 (19%)
Bilateral axillary involvement	0	12 (29%)	4 (21%)	0	16 (6.2%)
Activity throughout aorta	0	33 (80%)	12 (63%)	0	45 (18%)

Statistics are presented as *n* (%) if not otherwise specified. 1 Median (IQR). 2 Includes ultrasound of any artery. 3 Includes both CT angiography and contrast CT, given that the arterial wall was described. CRP C-reactive protein; ESR Erytrhcyte sedimentation rate; CT Computed tomography; MR Magnetic resonance; FDG-PET Fluorodeoxyglucose positron emission tomography; IQR Interquartile range.

**Figure 2:**
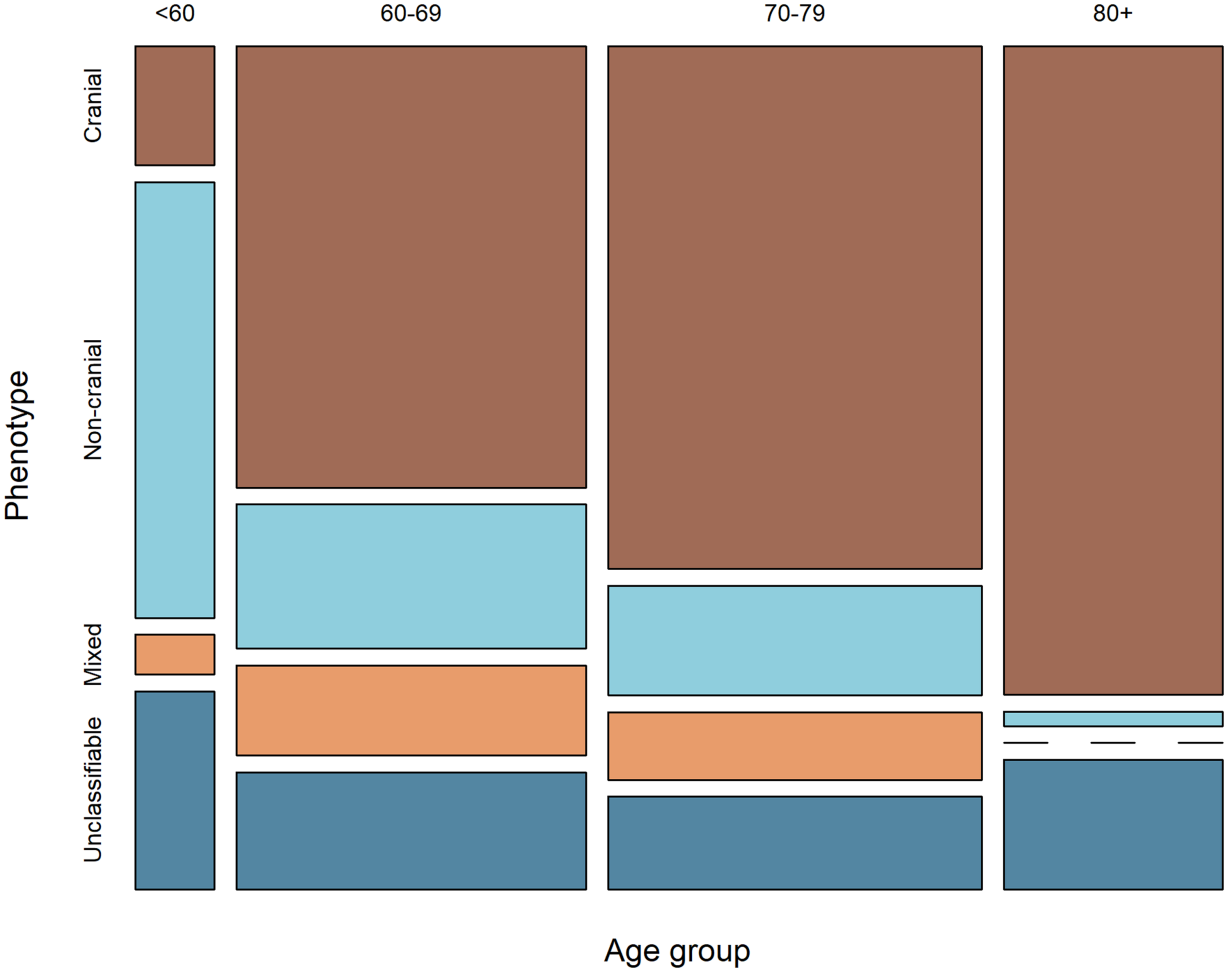
Mosaic plot showing the relationship between age-group and phenotype. The width of the columns indicates the proportion of patients within each age-group, while the height of the rectangles indicates the proportion of patients of each phenotype within each age-group.

The majority of patients under 60 years of age had non-cranial phenotype, while in the older age groups this proportion was lower (*p* < 0.001). The opposite was seen for cranial phenotype (Figure 2).

**Figure 3:**
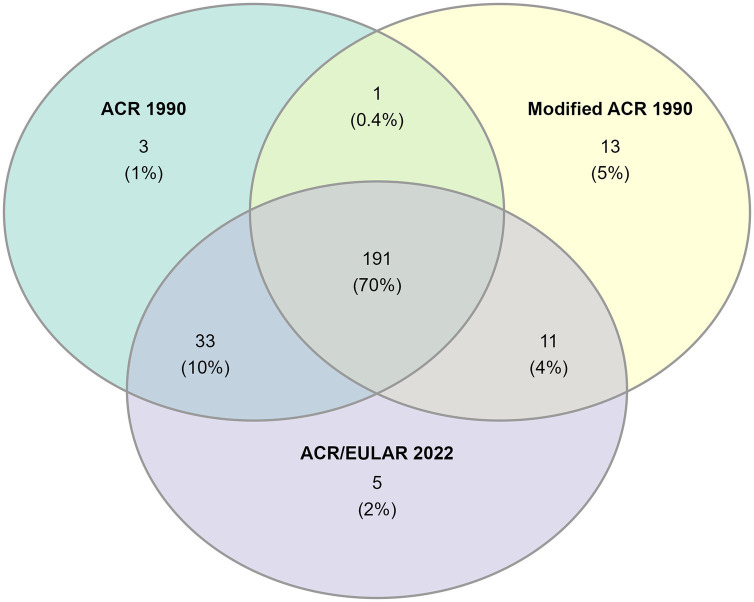
Venn-diagram showing the number of patients fulfilling each set of classification criteria.

191 (70%) patients fulfilled all three sets of classification criteria (Figure 3). Nearly all patients with cranial phenotype were captured by the classification criteria, and the modified ACR 1990 and ACR/EULAR 2022 captured all patients of mixed phenotype (Table 2). Capture of the non-cranial phenotype ranged from 49% (ACR 1990) to 90% (Modified ACR 1990) (Table 2).

**Table 2:** Fulfillment of classification criteria by phenotype.

	Phenotype				
	Cranial *N* = 159^1^	Non-cranial *N* = 41^1^	Mixed *N* = 19^1^	Unclassifiable *N* = 38^1^	Total *N* = 257^1^
ACR 1990	158 (99%)	20 (49%)	17 (89%)	33 (87%)	228 (89%)
Modified ACR 1990	159 (100%)	37 (90%)	19 (100%)	1 (2.6%)	216 (84%)
ACR/EULAR 2022	159 (100%)	26 (63%)	19 (100%)	36 (95%)	240 (93%)

^1^*n* (%) (column wise percentage).

**Figure 4:**
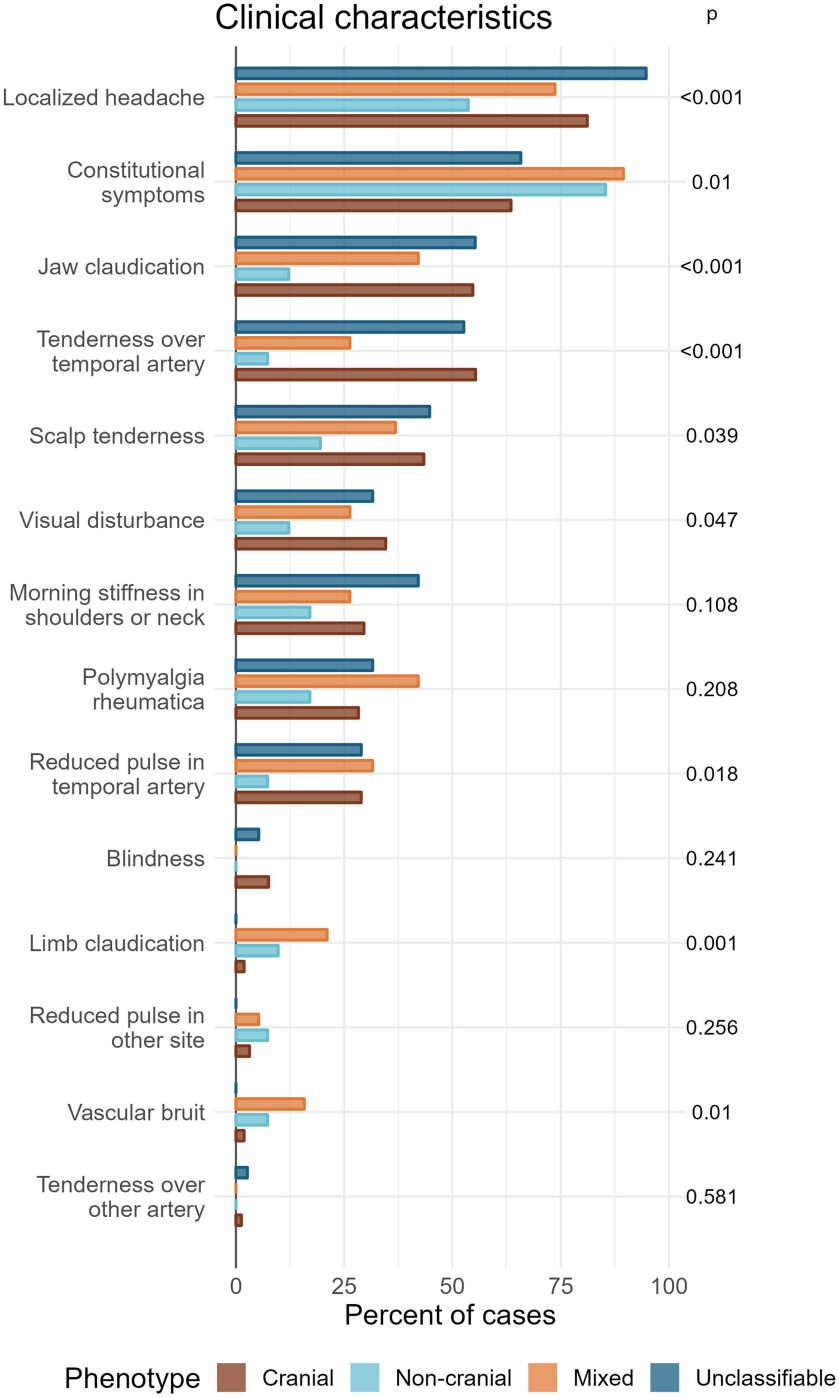
Grouped bar plot showing the percentage of patients of each phenotype expressing different clinical characteristics, ordered according to frequency of occurrence in total. *p*-values (unadjusted) are calculated by Chi-square test or Fisher’s test. Corrections for multiple testing gives a significance level of 0.0026 by the most conservative method (Bonferroni) or 0.018 by the less conservative method (Benjamini-Hochberg).

Overall, localized headache was the most common clinical feature (78% of patients) followed by constitutional symptoms (69%). All other clinical features were each present in less than 50% of the complete cohort (Figure 4).

Four features showed a significant association with phenotype after the conservative Bonferroni correction: localized headache, jaw claudication, and tenderness over temporal artery (*p* < 0.001), and limb claudication (*p* = 0.001). Another three features showed significance with the less conservative correction (Benjamini-Hochberg): Constitutional symptoms (*p* = 0.01), vascular bruit (*p* = 0.01), and reduced pulse in temporal artery (*p* = 0.018).

### Incidence estimates

**Figure 5:**
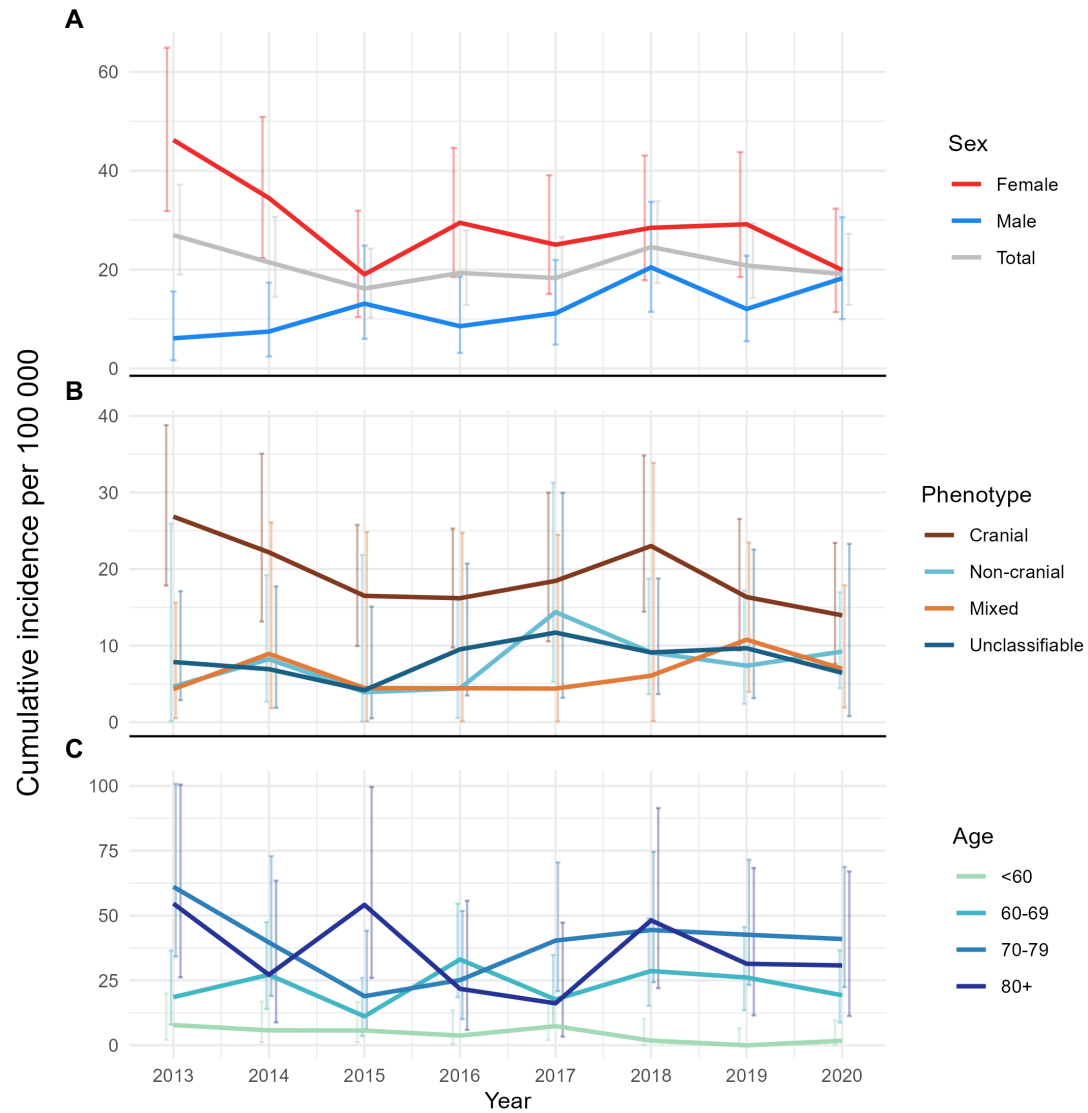
Line plots of annual cumulative incidence with error-bars showing the corresponding 95% confidence interval. **(A)** Overall and by sex, **(B)** by phenotype, and **(C)** by age-group.

The overall annual incidence during the study period was 20.7 (95% CI 18.2–23.5) per 100,000 persons aged 50 years or older. Figure 5 shows the overall as well as age-, sex- and phenotype-specific incidences during the study period. The cranial phenotype was predominating throughout the study period (Figure 5B). Incidence, as well as the variation in incidence, was lowest for patients below 60 years of age (Figure 5C).

## Discussion

In this large Norwegian GCA cohort, we found an overall incidence comparable to that shown by Brekke et al. for the predecessing time period in the same area (18). Incidence estimates are also comparable to other studies from Scandinavian countries (11, 25, 26), and the cohort is comparable to other studies regarding age and sex (11–13, 18). A Swedish study found a decreasing incidence of biopsy-confirmed GCA in the period 1997–2019, and the authors proposed that changes in the diagnostic work-up could be an explanation (27). A Danish study on GCA from 1996–2018 showed that the use of TAB declined while the use of diagnostic imaging increased (11). Our findings also reflect a change in the diagnostic work-up of GCA-patients. There is increased use of diagnostic imaging, but TAB remains a dominant diagnostic tool. These changes can be seen in conjunction with the observed changes in incidence, namely the decreasing tendency of the cranial phenotype and the increasing tendency of the non-cranial and mixed phenotypes. While the cranial phenotype was most prevalent overall, the non-cranial phenotype was significantly more common in patients under 60 years of age, in whom this was the most common presentation. Similar findings have been presented before (12, 13, 28).

Though headache was significantly more common in patients with cranial and unclassifiable phenotype, more than 50% of patients with non-cranial phenotype also had new localized headache. Some other studies have reported similar findings (10, 29, 30). Constitutional symptoms were more common in non-cranial and mixed phenotype, with borderline significance (depending on method of multiple-testing correction). For the non-cranial phenotype, the occurrence of all other clinical features each were < 25%, underlining the difficulties clinicians may face in the diagnostic process for these patients. The low occurrence of “hallmark” GCA-features could explain why patients of non-cranial phenotype have longer diagnostic delays. This supports the current recommendations regarding examination of non-cranial arteries in the work-up of GCA (5).

The present study is one of the first to systematically analyze the distribution of GCA phenotypes beyond the binary division between cranial and non-cranial phenotypes. A GCA-cohort based on the ACR/EULAR-endorsed study to develop Diagnostic and Classification Criteria for Vasculitis (DCVAS) reported a phenotype distribution comparable to our findings (10). A small study using CT angiography to examine newly diagnosed GCA-patients found that two thirds had involvement of non-cranial arteries, whereas a study based on FDG-PET/CT-results showed involvement of non-cranial arteries in 83% of GCA-patients (31, 32). A Norwegian study using vascular ultrasound showed involvement of non-cranial vessels in 93 of 133 patients (70%) (16). A major difference between these studies and our study is the study design, with the possibility of missing imaging data for some patients in our study causing a possible underestimation of non-cranial involvement. A retrospective study from Japan found that 18 out of 36 (50%) patients had involvement of non-cranial arteries (33), while another retrospective study from New Zealand found documented involvement of non-cranial arteries in only 10 out of 142 (7%) patients (29).

### Strengths and limitations

Limitations of our study are largely due to the observational retrospective design. There is a risk of missing data and wrongfully recorded data. This is especially relevant for the group of patients excluded based on a diagnosis of polymyalgia rheumatica, alone. It is possible that some of these are misdiagnosed GCA-patients, and this could result in underestimation of the true incidence. However, we did a thorough review of the patient records and included patients only when sufficient information was available.

A major strength of our cohort is its completeness. We screened patient records of all patients who received an ICD-code applicable for GCA and included only validated GCA cases. Another strength is the objectively defined phenotypes based on results of biopsy and imaging diagnostics.

Our high exclusion proportion suggests a discrepancy between medical coding and clinical evaluation. In particular, we noticed a practice of using a disease-related ICD-code for diagnostic procedures, specifically TAB. The validation process for our cohort incorporating the use of classification criteria, however, gives a strong basis for a cohort of correctly identified GCA-patients.

A major problem when comparing studies on non-cranial GCA has been a lack of a standardized classification for GCA phenotypes. We believe that our classification can be an example for future studies as it encompasses a broader spectrum better reflecting the GCA patient population.

## Conclusion

In conclusion, we found that the overall incidence for GCA in Western Norway remained stable from 2013 to 2020, and was comparable to earlier reports from the same region. The cranial phenotype dominated although the non-cranial phenotype was more common in patients under 60 years of age. The diagnostic delay was longer in patients with the non-cranial versus cranial phenotype, indicating a need for examination of non-cranial arteries when suspecting GCA.

## Data availability statement

The datasets presented in this article are not readily available because the data contains identifyable information. Requests to access the datasets should be directed to hans.kristian.skaug@hsr.as.

## Ethics statement

The studies involving humans were approved by Regional Committee for Medical and Health Research Ethics (REK Vest). The studies were conducted in accordance with the local legislation and institutional requirements. Written informed consent for participation was not required from the participants or the participants’ legal guardians/next of kin in accordance with the national legislation and institutional requirements.

## Author contributions

HS: Conceptualization, Data curation, Formal analysis, Funding acquisition, Investigation, Methodology, Validation, Visualization, Writing – original draft, Writing – review & editing. BF: Conceptualization, Methodology, Project administration, Supervision, Writing – review & editing. JA: Data curation, Formal analysis, Writing – review & editing. AD: Writing – review & editing. GM: Writing – review & editing. LB: Conceptualization, Funding acquisition, Methodology, Validation, Writing – review & editing, Supervision.

## Footnotes

^1^ www.ssb.no
